# Nutritional risk screening 2002 scale and subsequent risk of stroke-associated infection in ischemic stroke: The REMISE study

**DOI:** 10.3389/fnut.2022.895803

**Published:** 2022-09-09

**Authors:** Xiaoli Chen, Dongze Li, Yi Liu, Ling Zhu, Yu Jia, Yongli Gao

**Affiliations:** ^1^Department of Emergency Medicine and West China School of Nursing, West China Hospital, Sichuan University, Chengdu, China; ^2^Department of General Practice and National Clinical Research Center for Geriatrics, West China Hospital, Sichuan University, Chengdu, China

**Keywords:** ischemic stroke, stroke-associated infection, NRS-2002, malnutrition, multicenter

## Abstract

**Background and aim:**

Stroke-associated infection (SAI) is a common and serious complication in patients with IS. This study aimed to evaluate the impact of nutritional status at admission assessed on SAI, explore the predictive value of the Nutritional Risk Screening 2002 (NRS-2002 for SAI.

**Methods:**

This study included patients with IS who were admitted to five major hospitals in Chengdu from January 2017 to February 2019. The nutritional status was assessed using the NRS-2002 tool. Logistic regression analysis was performed to explore the predictive value of NRS-2002 for SAI.

**Results:**

A total of 594 patients with IS were included in this study; among them, 215 (36.20%) patients were at risk of malnutrition, and 216 (36.36%) patients developed SAI. The area under the curve of the NRS-2002 scores was smaller than A2DS2 (0.644 vs. 0.779), and NRS-2002 improved the predictive values of the A^2^DS^2^ score(Age, Atrial fibrillation, Dysphagia, Sex, Stroke Severity) for SAI (*P* < 0.001). Logistic regression analysis showed that patients with NRS-2002 score ≥ 3 had significantly higher risks of SAI (NRS-2002: odds ratios (OR) = 1.450, 95% confidence interval (CI): 1.184–1.692, *P* < 0.001).

**Conclusion:**

NRS-2002 is a useful and simple tool for identifying the risk of SAI. Malnutrition is related to the development of SAI. Malnourished patients with stroke may benefit from further nutritional supplements and management.

## Introduction

Ischemic stroke (IS) is the main type of stroke in clinical practice, accounting for approximately 69.6% of all strokes (1), with the characteristics of high morbidity, high disability rate, high mortality, and high recurrence rate (2). The occurrence of serious complications is the most important cause of death and disability in patients with IS. Stroke-associated infection (SAI) is one of the extremely common and serious complications, with an incidence of 23–65% (3, 4). SAI not only prolongs the length of hospitalization and increases the economic burden but also is closely related to the increase in severe disability and mortality (5). Therefore, early evaluation and prevention of SAI is considered an important part of stroke diagnosis and treatment. The current diagnosis of SAI is mainly based on the inflammatory marker procalcitonin (PCT) and computed tomography (CT) scan, but both indicators only appear clinically after infection. Therefore, it is of great significance to find effective assessment tools for the early prevention and treatment of SAI.

Malnutrition is a common condition in patients with stroke. The prevalence of malnutrition is up to 62% in patients with stroke (6) due to reduced mobility, cognitive impairments, and dysphagia (7). Malnutrition may worsen clinical outcomes and increase mortality (8–10). The mortality rate of malnourished patients with stroke was significantly higher. Research has revealed that malnutrition leads to immune dysfunction (11), resulting in reduced resistance to bacteria and pathogens, and heightens drug resistance, which in turn leads to an increased probability of infection. It was noted that patients with stroke are prone to become immunocompromised due to malnutrition, thus increasing the probability of SAI.

The Nutritional Risk Screening 2002 (NRS-2002) tool (12, 13) is a nutritional assessment tool that is widely used in clinical applications. The effectiveness of this tool has been confirmed by a large number of clinical studies (14, 15). Kyle et al. found that the NRS-2002 tool had higher sensitivity and specificity than other nutritional assessment tools (16). Nutrition may be a reliable indicator for predicting infection. Therefore, nutritional screening tools can be used for the early risk assessment of SAI. To the best of our knowledge, no studies have shown that nutritional risk assessment tools can be used for the early identification of SAI.

This study aimed to evaluate the nutritional status of patients with IS using the NRS-2002 tool and explore its correlation with SAI occurrence to screen for high risk of SAI and early clinical intervention to improve the prognosis of patients with IS.

## Materials and methods

### Study design

This is a retrospective multicenter study based on recovery trajectory data to assess whether NRS-2002 can predict the incidence of SAI in patients with IS. Patients were recruited from the stroke centers of five hospitals. This study was registered at www.chictr.org.cn (Identifier: ChiCTR2100052025) and conducted in accordance with the Helsinki Declaration. The research plan was approved by the Human Ethics Committee of West China Hospital of Sichuan University. Informed consent was obtained from each participant.

### Study population

Patients with acute stroke admitted to the Emergency Stroke Center of five major hospitals in Chengdu, Sichuan, from January 2017 to February 2019 were retrospectively included in this study. The inclusion criteria were as follows: (1) in line with diagnostic criteria for IS (17); (2) patients who were admitted to hospitals within 24 h after onset and diagnosed with cerebral infarction by head CT or head magnetic resonance imaging (MRI); (3) age ≥ 18 years. The exclusion criteria were as follows: (1) severe liver, kidney, and hematological diseases; (2) previous history of stroke and legacy sequelae; (3) cancer history or immunosuppressive therapy; (4) incomplete clinical data.

### Data collection and measures

The following data were retrieved from the database of retrospective multicenter studies on IS: demographic statistics and characteristic information, vital signs, medical history, drugs, laboratory tests, hospitalization, and adverse outcomes. In this study, blood cells were counted using a hematology analysis system (LH750, Beckman Coulter, Brea, CA). Blood biochemical tests were performed using an Architect C16000 analyzer (Abbott Diagnostics, Dallas, TX, USA).

The A^2^DS^2^ score (Age, Atrial fibrillation, Dysphagia, Sex, Stroke Severity) was calculated (age ≥ 75 years = 1; male sex = 1; atrial fibrillation = 1; dysphagia = 2; stroke severity: National Institutes of Health Stroke Scale (NIHSS) score ≥ 5 = 3) (18), which is a validated screening tool for stroke-associated pneumonia, and the range of A^2^DS^2^ score was from 0 to 10, and 0–4 points for low risk and 5–10 points for high risk. The NIHSS (19) which can assess stroke severity, was administered at admission and discharge. The range of the NIHSS scores was from 0 to 42.

### Malnutrition screening tools

The NRS-2002 tool (12) was used to evaluate the nutritional status of patients by trained nurses. The NRS-2002 score ranges from 0 to 7 and consists of three components: (1) disease severity (reflecting increased nutritional needs), which ranges from 0 to 3 based on the patient's comorbidities and medical history; (2) nutritional impairment status, which relates to BMI, body weight, and food, which ranges from 0 to 3; (3) age, with one point for patients ≥ 70 years old. According to European Society for Parental and Enteral Nutrition(ESPEN) guidelines for malnutrition diagnosis (12), patients with NRS-2002 scores ≥3 were identified as malnourished.

### Outcomes

The main outcome was SAI during hospitalization. According to the revised standard definition of the Centers for Disease Control and Prevention based on electronic medical records (20), SAI was defined as an infection in the 1st week after stroke, mainly including the following three types: pneumonia, urinary tract infection, and other infections. All infections were reviewed and verified by experienced clinical doctors.

### Statistical analysis

Parametric continuous variables were represented by mean ± standard deviation, and nonparametric continuous variables were represented by the median of the quartile interval. Categorical variables were reported in the form of frequencies and percentages. The *t*-test was used for comparison between groups with parametric characteristics, and the Mann–Whitney U test was used for comparison between groups with non-parametric characteristics. A chi-square test or Fisher's exact test was used to compare categorical variables.

Logistic regression analysis was performed to analyze the relationship between malnutrition and SAI. The logistic regression model was adjusted for demographic variables (sex, age, smoking, and drinking), physiological variables (body mass index and blood pressure), laboratory tests (hemoglobin, urea nitrogen, and creatinine), and chronic diseases (hypertension, diabetes, and hyperlipidemia) to further determine whether these relationships were independent of risk factors. In the model, the patients were divided into groups according to age (<65 years vs. ≥65 years), sex (male vs. female), hypertension (yes vs. no), diabetes (yes vs. no), treatment (conservative vs. thrombolysis or thrombectomy), and NIHSS score (≤ 7 vs. >7). The area under the receiver operating characteristic (ROC) curve and continuous net reclassification improvement (NRI) were established to evaluate the predictive ability of NRS-2002 and A^2^DS^2^ for SAI.

The significance level was set at 0.05. All statistical analyses were performed using SPSS version 26.0 (IBM Corp, Armonk, NY, USA) and R software 3.5.0 (Vienna, Austria).

## Results

### Baseline patient characteristics

Finally, after an overview of the inclusion and exclusion criteria, 594 patients were included in this study. The average age was 58.79 ± 11.97 years. Of these participants, 378 (63.6%) were male. In addition, 215 (36.20%) patients were at risk of malnutrition (NRS-2000≥3). A total of 216 (36.36%) patients developed SAI during hospitalization. At admission, patients with NRS-2002 score ≥3 were found to be older, have a lower Barthel index and hemoglobin, have a higher D-dimer, and high-density lipoprotein, and have a higher NIHSS score and A^2^DS^2^ score. The baseline characteristics of the patients are presented in Table 1. Table 1 show the baseline characteristics of the patients.

**Table 1:** Baseline patient characteristics and nutritional risk score 2002 in patients with ischemic stroke.

**Characteristic**	**NRS-2002 <3** ***N* = 379**	**NRS-2002 ≥3** ***N* = 215**	***P***
Demographic variables			
Age, years	58.79 ± 11.97	76.49 ± 9.08	<0.001
Males, *n* (%)	264 (70)	114 (53)	<0.001
Smoking, *n* (%)	179 (47)	70 (33)	<0.001
Drinking, *n* (%)	151 (40)	26 (12)	<0.001
Chronic medical conditions			
Hypertension, *n* (%)	223 (59)	137 (64)	0.242
Diabetes, *n* (%)	104 (27)	54 (25)	0.538
Hyperlipidemia, *n* (%)	49 (13)	10 (5)	0.001
Coronary heart disease, *n* (%)	31 (8)	17 (8)	0.907
History of cancer, *n* (%)	17 (4)	12 (6)	0.551
Physiological and lab variables			
BMI, kg/m^2^	24.39 ± 3.02	22.94 ± 4.91	<0.001
Admission SBP, mmHg	144.09 ± 25.45	148.02 ± 26.06	0.075
Admission DBP, mmHg	88.91 ± 16.22	86.73 ± 17.91	0.132
Heart rate,/min	82.39 ± 16.22	81.28 ± 18.85	0.618
Temperature, °C	36.49 ± 0.27	36.51 ± 0.32	0.36
Hemoglobin, g/L	139.82 ± 19.67	130.00 ± 17.132	<0.001
WBC, *10^9^/L	7.98 ± 2.99	7.78 ± 2.80	0.451
Neutrophil, *10^9^/L	71.11 ± 11.84	76.98 ± 36.71	0.005
Platelet count, *10^9^/L	189.64 ± 72.57	169.31 ± 61.92	0.001
Albumin, g/l	70.36 ± 5.77	70.29 ± 6.17	0.117
D-dimer, mg/L	0.41 (0.21–.23)	1.04 (0.52–2.46)	<0.001
Fibrinogen, g/L	2.91 ± 0.98	3.24 ± 1.11	0.204
Blood glucose, mmol/L	7.73 ± 3.19	8.17 ± 3.30	0.11
Creatinine, μmol/L	72.0 (63.0–84.0)	75.0 (62.0–92.0)	0.012
BUN, mmol/L	5.48 (4.30–6.50)	5.80 (4.60–7.70)	0.003
Triglycerides, mmol/L	1.79 ± 1.46	1.41 ± 0.93	0.001
Total cholesterol, mmol/L	4.88 ± 3.91	5.19 ± 5.96	0.432
HDL, mmol/L	1.19 ± 0.37	1.37 ± 0.47	<0.001
LDL, mmol/L	2.42 (1.87–3.06)	2.42 (1.88–3.06)	0.472
Risk scores			
NIHSS score	6 (2–13)	10 (4–17)	<0.001
Barthel index	55 (20–75)	25 (10–50)	<0.001
A^2^DS^2^ score	4 (1–4)	5 (3–6)	<0.001

### Relationship between NRS-2002 scores and SAI

**Figure 1:**
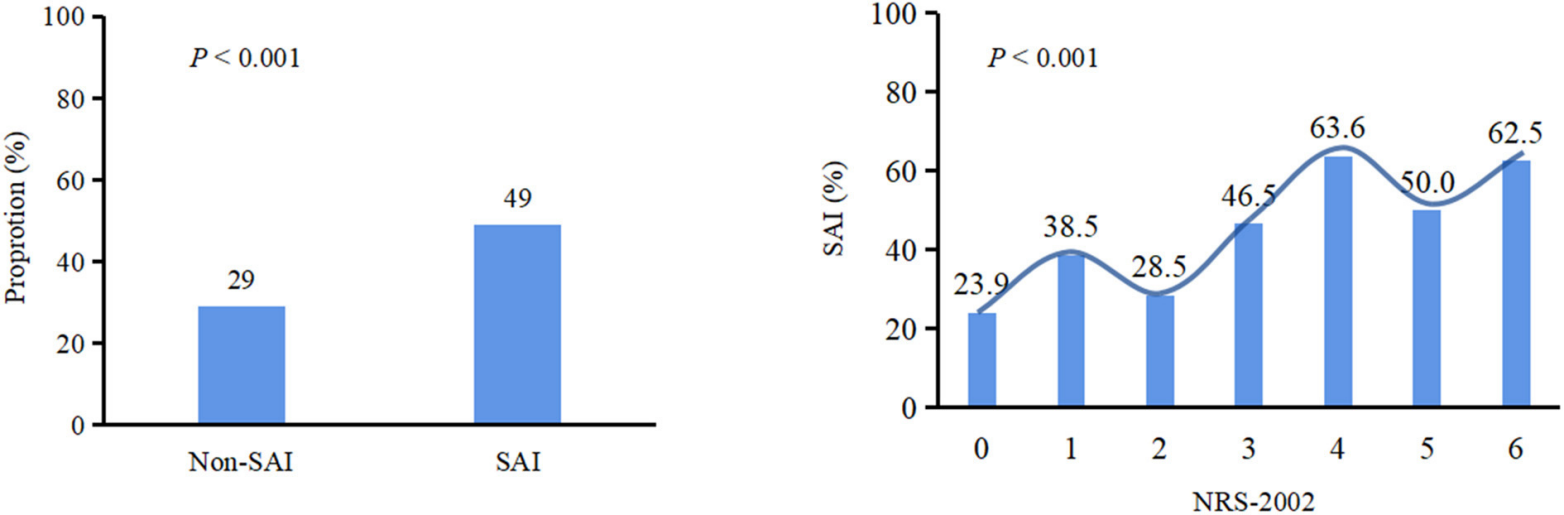
Chi square test was used to compare categorical variables. The proportion of patients with malnutrition (NRS-2002≥3) in SAI is higher than that in non-SAI (*P* < 0.001). Patients with NRS-2002 score ≥3 were more likely to develop SAI (*P* < 0.001). SAI, Stroke-associated infection;NRS-2002, Nutritional Risk Screening 2002.

**Figure 2:**
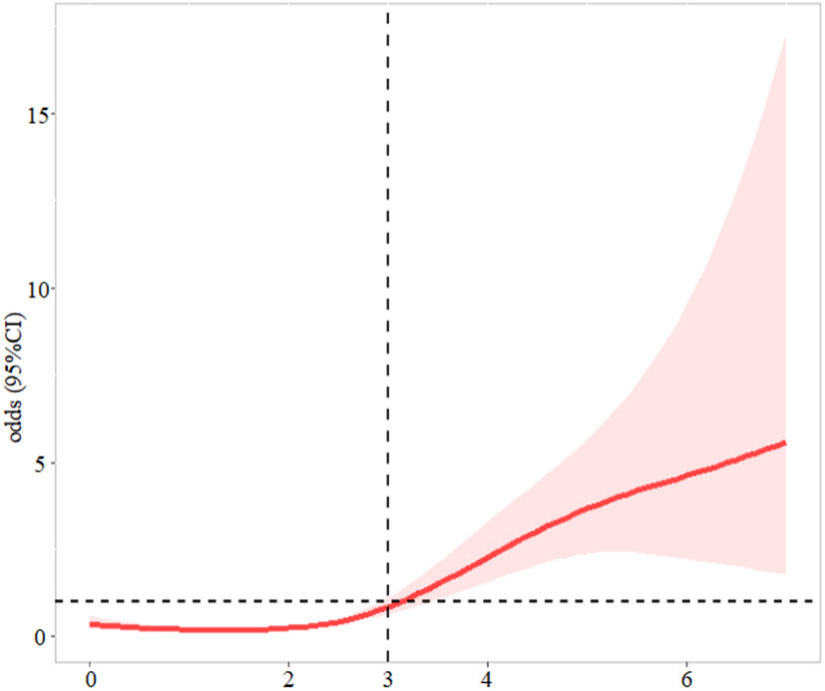
The association between NRS-2002 and odds ratio for stroke-associated infection. The solid line indicates the point estimate, and the shaded area is the 95% CI. Models were adjusted by by age, sex, smoking (never, former, current), drinking (never, former, current), body mass index, systolic blood pressure, smoking, drinking,.hemoglobin, urea nitrogen, and creatinine.

Patients with NRS-2002 score ≥3 were more likely to develop SAI (*P* < 0.001) (Figure 1). In the logistic regression model (Table 2), after adjusting for demographic variables, physical examination, laboratory testing, and chronic medical conditions, patients with malnutrition risk (assessed by NRS-2002) or neurological deficit (assessed by NIHSS) had significantly higher risks of SAI (NRS-2002: odds ratio (OR) = 1.350, 95% confidence interval (CI): 1.144–1.592, *P* < 0.001; NIHSS: OR = 1.142, 95% CI: 1.098–1.189, *P* < 0.001). Restricted cubic spline analyses demonstrated continuous relationships between NRS-2002 and odds for SAI (Figure 2).

**Table 2:** Adjusted ORs (95% CI) for the association of NRS-2002, NIHSS, and A^2^DS^2^ scores with the incidence of stroke-associated infection.

**Variables**	**Unadjusted**		**Model 1**		**Model 2**	
	**OR (95% CI)**	***P*-trend**	**OR (95% CI)**	***P-*trend**	**OR (95% CI)**	***P*-trend**
NRS-2002	1.358 (1.186,1.554)	<0.001	1.371 (1.176,1.613)	<0.001	1.350 (1.144,1.592)	<0.001
NIHSS	1.145 (1.111,1.181)	<0.001	1.163 (1.120,1.208)	<0.001	1.142 (1.098,1.189)	<0.001
A^2^DS^2^	1.621 (1.467,1.791)	<0.001	1.590 (1.416,1.784)	<0.001	1.161 (1.117,1.207)	<0.001

Model 1: adjusted by age, sex, smoking (never, former, current), drinking (never, former, current), body mass index, systolic blood pressure, smoking, and drinking.

Model 2: adjusted by model 1 plus hemoglobin, urea nitrogen, creatinine.

OR, odds ratio; CI, confidence interval.

### Predictive value of NRS-2002 for SAI

According to ROC curve analysis, the area under the curve (AUC) of NRS-2002 and A^2^DS^2^ for SAI was 0.644 (95% CI: 0.588–0.700, *P* < 0.001) and 0.779 (95% CI: 0.735–0.823, *P* < 0.001), respectively. A^2^DS^2^ combined with NRS-2002 achieved a higher AUC (0.822, 95% CI:0.793- 0.862) than A^2^DS^2^ alone (*P* < 0.001). Additionally, NRS-2002 combined with A^2^DS^2^ achieved additional predictive values for SAI beyond A^2^DS^2^ according to the NRI analysis (NRI = 0.095, 95% CI: 0.048–0.139, *P* < 0.001) (Table 3, Supplementary Figure 1).

**Table 3:** Predictive value of the NRS-2002, NIHSS and A^2^DS^2^ for stroke-associated infection.

**Variables**	**AUROC (95% CI)**	**P for AUROC**	**P for AUROC comparison**	**NRI (95% CI)**	**P for NRI**
NRS-2002	0.644(0.588, 0.700)	<0.001	<0.001	−0.182 (−0.121, −0.259)	<0.001
A^2^DS^2^	0.779(0.735, 0.823)	<0.001	Ref.	Ref.	-
NRS-2002 plus A^2^DS^2^	0.822(0.793, 0.862)	<0.001	<0.001	0.095 (0.048, 0.139)	<0.001

CI, confidence interval; AUROC, area under the receiver operating characteristic curve; NRI, net reclassification improvement; NIHSS, National Institutes of Health Stroke Rating Scale; NRS-2002, Nutritional Risk Screening 2002; A^2^DS^2^, Age, Atrial fibrillation, Dysphagia, Sex, Stroke Severity.

An NRS-2002 score ≥5 had optimal specificity (95.69%) to predict SAI (Table 4).

**Table 4:** The predictive value of nutritional risk screening 2002 for stroke associated infection in patients with ischemic stroke.

**NRS-2002**	**Sensitivity, %**	**Specificity, %**	**Accuracy, %**	**PPV, %**	**NPV, %**
≥0	100.00	0.00	36.36	36.36	—
≥1	97.66	14.74	44.61	39.21	91.80
≥2	90.27	23.37	48.82	41.98	79.63
≥3	62.96	71.16	68.18	55.51	77.08
≥4	34.01	92.44	73.06	69.07	73.84
≥5	19.92	95.69	64.31	76.56	62.83
≥6	10.33	94.75	64.48	52.38	65.40
≥7	0.93	100.00	63.97	100.00	63.85

### Subgroup analysis

**Figure 3:**
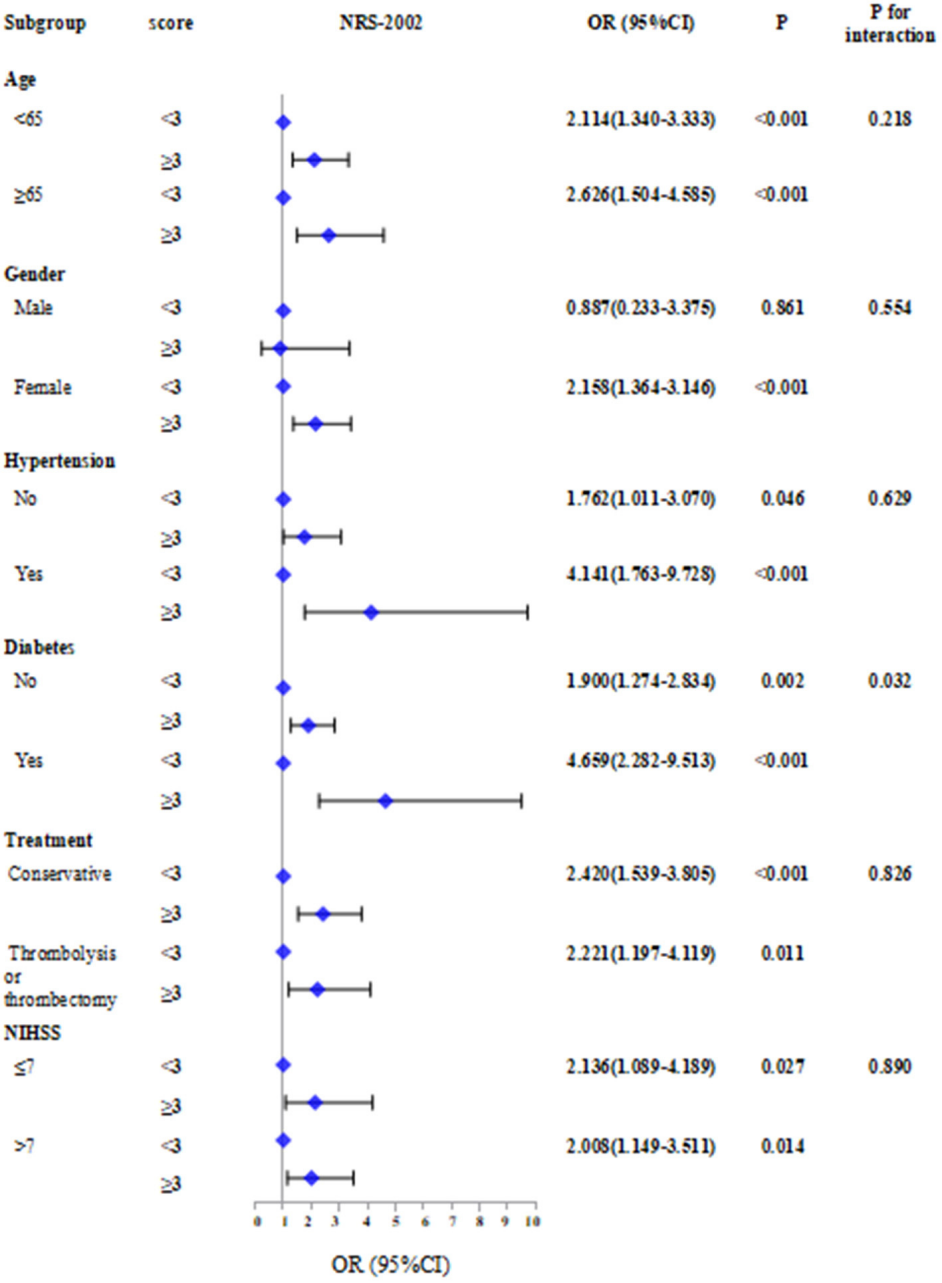
Subgroup analyses stratified by age, sex, hypertension, diabetes, treatment and NIHSS score.The absent of malnutrition group was used as reference in Logistic regression models.

Subgroup analyses stratified by age, sex, hypertension, diabetes, treatment, and NIHSS score were performed (Figure 3).

The results showed that patients with NRS-2002 score ≥3 combined with other factors including age ≥ 65 (OR = 2.626, 95% CI: 1.504–4.585, *P* < 0.001), female (OR = 2.158, 95% CI: 1.364–3.146, *P* < 0.001), hypertension (OR = 4.141, 95% CI: 1.763–9.728, *P* < 0.001), diabetes (OR = 4.659, 95% CI: 2.282–9.513, *P* < 0.001), and NIHSS score >7 (OR = 2.008, 95% CI: 1.149–3.511, *P* = 0.014) have a higher risk of SAI. In contrast, the risk of SAI associated with NRS-2002 was significantly stronger for patients with diabetes than for those without diabetes (*P* for interaction: 0.032).

## Discussion

Stroke is the second major cause of death worldwide and the leading cause of death and disability in China (21). The clinical data of 594 patients with IS were analyzed; among them, 31.19% had malnutrition, and 36.36% had SAI. The results show that malnutrition and malnutrition risk are risk factors for SAI and using NRS-2002 to predict SAI is effective. The comprehensive evaluation recommended early nutritional screening as an effective measure to reduce the incidence of SAI.

Recently, malnutrition has been reported to increase the incidence of infections in patients with stroke during rehabilitation and delay the recovery of motor nerve function (22, 23). Studies have shown that malnutrition can affect the size of organs, hormones, and cytokine levels and even affect the immune cells' function (24). Malnutrition is associated with immunosuppression, leading to increased susceptibility to infection. Furthermore, studies have reported that patients with IS at risk of malnutrition are 3.40 and 4.08 times more likely to develop infection complications than those with normal nutrition, respectively (25). Early nutritional support for malnourished patients with IS through nutritional assessment using NRS 2002 can help improve the prognosis. It is recommended to use NRS-2002 to guide nutritional support, which can predict short-term and long-term outcomes (26). To the best of our knowledge, this is the first study that uses the NRS-2002 tool to predict the occurrence of SAI in patients with IS. The factors that constitute the index may explain the relationship between the malnutrition index and SAI. Patients with NRS-2002 score ≥3 were more likely to develop SAI (*P* < 0.001). The logistic regression model showed that patients with malnutrition risk (assessed by NRS-2002) or neurological deficit (assessed by NIHSS) had significantly higher risks of SAI, which is consistent with previous studies (27, 28). The higher the NIHSS score, the more serious the neurological deficit, the higher the risk of bed rest, and the more prone to follow pneumonia and aspiration pneumonia caused by gastroesophageal reflux (29).

In this study, we validated the diagnostic value of NRS-2002 for SAI in patients with IS. Our data showed that NRS-2002 could predict the SAI. Moreover, considering sensitivity, specificity, and AUC, the results showed that NRS-2002 combined with A^2^DS^2^ can optimize the prediction effect of SAI. According to ROC curve analysis, the AUC of NRS-2002, A^2^DS^2^, and A^2^DS^2^ combined with NRS-2002 was 0.644, 0.779, and 0.822, respectively. Based on the NRI analysis, NRS-2002 combined with A^2^DS^2^ achieved additional predictive values for SAI beyond A^2^DS^2^ (NRI = 0.095, 95% CI: 0.048–0.139, *P* < 0.001). Because the A^2^DS^2^ does not include the evaluation of nutrition and malnutrition increases the SAI incidence, it is meaningful to include the evaluation of nutrition in the tool for predicting SAI. Subgroup analysis shows that NRS-2002 predicts a better value in SAI for patients with diabetes. In hyperglycemic patients, insulin is relatively insufficient due to insulin resistance, and glucose utilization is reduced. This increases the nutritional risk of the patient. The risk of malnutrition assessed using NRS-2002 is related to the development of SAI. NRS-2002 may be a useful and simple tool for identifying SAI.

This study also has some limitations. First, this is a retrospective study and needs to be confirmed by prospective studies. Second, the evaluation results of the scale were not compared with the infection biomarkers, and the evaluation results were not further verified. Third, the study failed to use NRS-2002 for continuity assessment during hospitalization.

IS is a common disease in clinical neurology. Generally, early nutritional screening should be part of the standard clinical treatment management for patients with IS. It is necessary to check the nutritional status and risk factors for patients with IS. The risk of malnutrition assessed using the NRS-2002 score is related to the development of SAI. NRS-2002 may be a useful and simple tool for identifying SAI. Patients with malnutrition and malnutrition risk should be given appropriate nutritional management as soon as possible to help reduce infection complications, improve prognosis, and reduce medical costs. However, how to provide nutritional supplements and management for malnourished patients with stroke is still a key issue faced by clinicians. Thus, further research is needed to explore an effective approach to address malnutrition in patients with IS.

## Data availability statement

The original contributions presented in the study are included in the article/Supplementary material, further inquiries can be directed to the corresponding author/s.

## Ethics statement

The studies involving human participants were reviewed and approved by the Human Ethics Committee of West China Hospital of Sichuan University. The patients/participants provided their written informed consent to participate in this study.

## Author contributions

XC, DL, and YG conceived the study design and interpreted the data and drafted the manuscript. XC, YJ, YL, and LZ collected the epidemiological and clinical data. XC, YJ, and YL summarized the data and performed the statistical analysis. All the authors have accepted responsibility for the entire content of this submitted manuscript and approved submission.

## Funding

This work was supported financially by grants from Sichuan Science and Technology Program (No. 2022YFS0279, 2021YFQ0062, and 2022JDRC0148), Sichuan University West China Nursing Discipline Development Special Fund Project (HXHL20017 and HXHL21016).

## Conflict of interest

The authors declare that the research was conducted in the absence of any commercial or financial relationships that could be construed as a potential conflict of interest.

## Publisher's note

All claims expressed in this article are solely those of the authors and do not necessarily represent those of their affiliated organizations, or those of the publisher, the editors and the reviewers. Any product that may be evaluated in this article, or claim that may be made by its manufacturer, is not guaranteed or endorsed by the publisher.

## Supplementary material

Supplementary Figure 1 Receiver operating characteristic curve for NRS-2002 and A^2^DS^2^ of the SAP.
