# Supplementary figures and images for: Nutritional risk screening 2002 scale and subsequent risk of stroke-associated infection in ischemic stroke: The REMISE study
Source: Front Nutr. 2022 Sep 9;9:895803. doi: 10.3389/fnut.2022.895803 (PMC9505510; doi:10.3389/fnut.2022.895803)

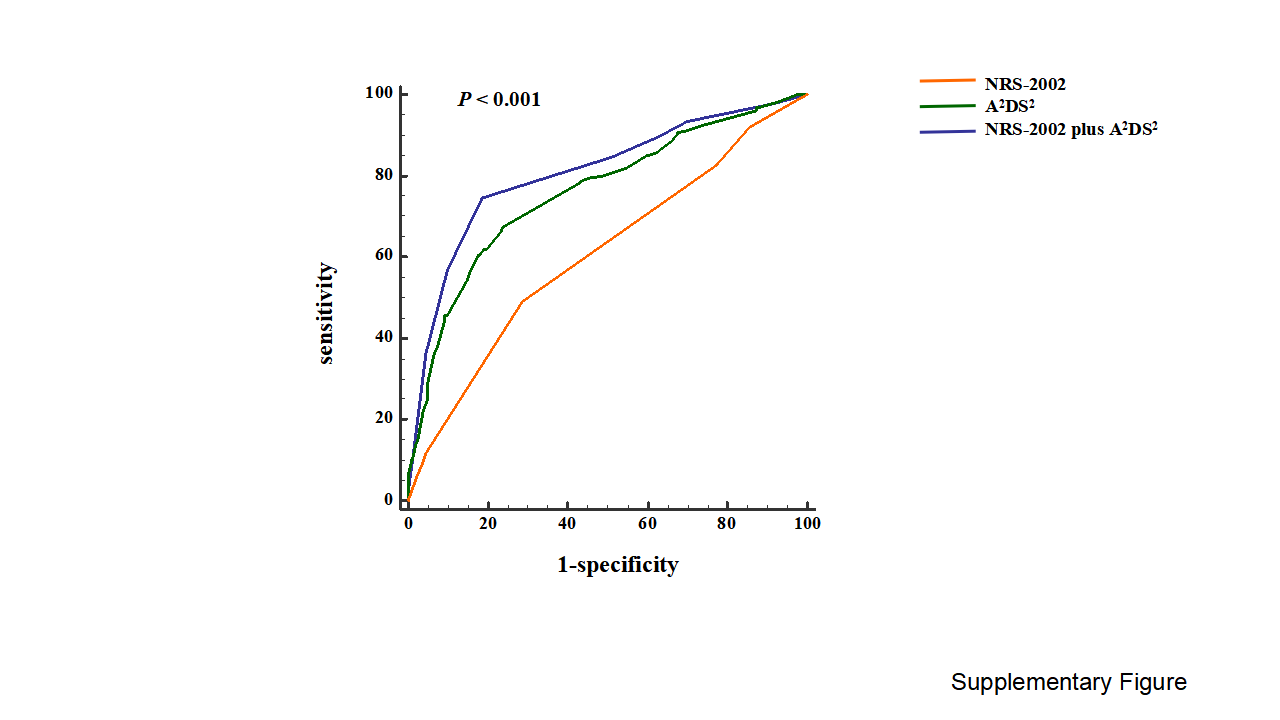

Supplement: Supplementary Figure 1 — Receiver operating characteristic curve for NRS-2002 and A2DS2 of the SAP. [file Image_1.tif]
